# Supplementary material for: COVID-19 and excess mortality in the United States: A county-level analysis
Source: PLoS Med. 2021 May 20;18(5):e1003571. doi: 10.1371/journal.pmed.1003571 (PMC8136644; doi:10.1371/journal.pmed.1003571)
Supplement: S1 Fig — (PDF) [file pmed.1003571.s001.pdf]

S1 Fig. Flowchart Detailing Sample Exclusions

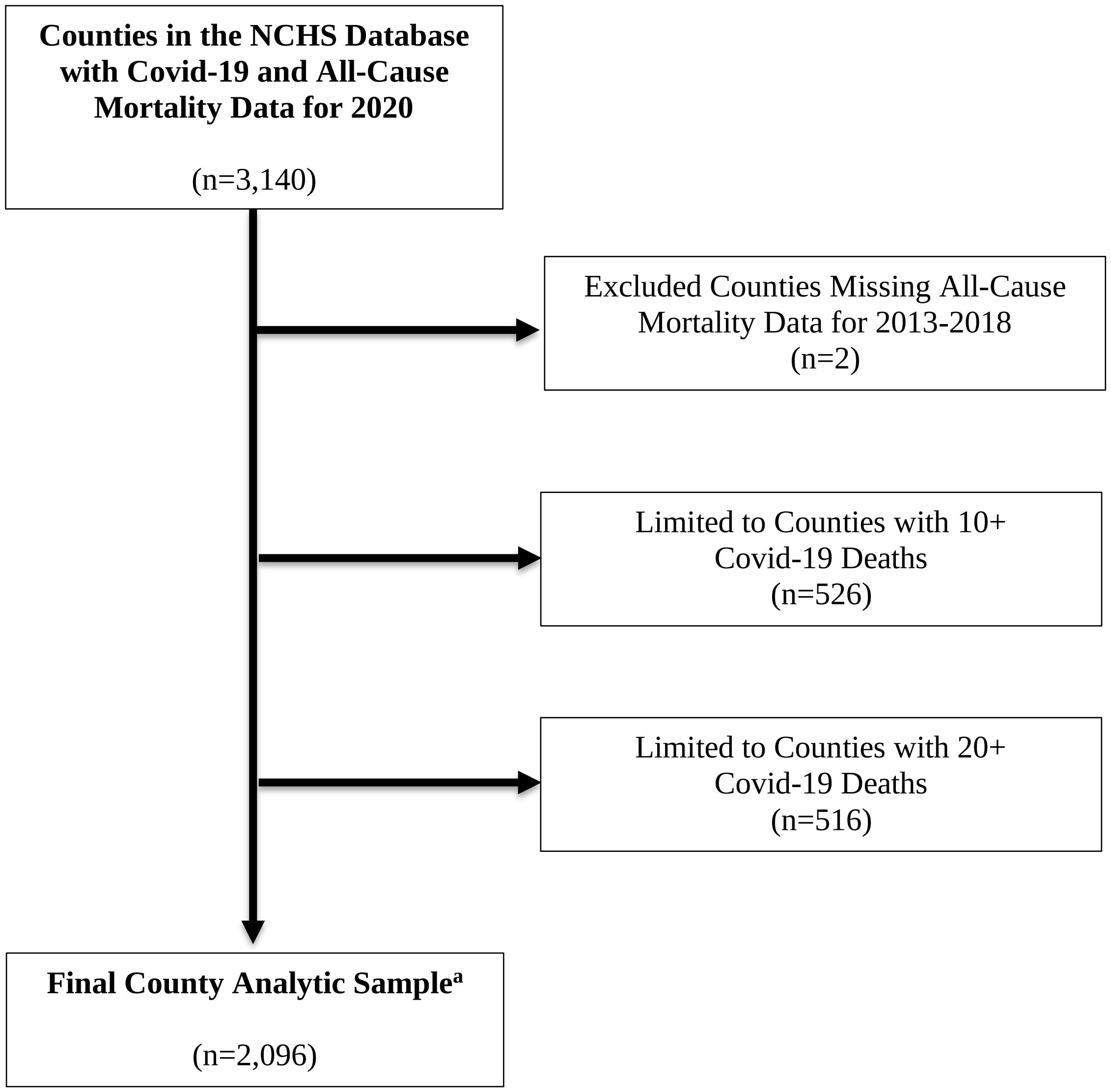

a. Counties had complete data for each of the sociodemographic and health characteristics we examined.
