# Supplementary material for: COVID-19 and excess mortality in the United States: A county-level analysis
Source: PLoS Med. 2021 May 20;18(5):e1003571. doi: 10.1371/journal.pmed.1003571 (PMC8136644; doi:10.1371/journal.pmed.1003571)
Supplement: S2 Fig — (PDF) [file pmed.1003571.s002.pdf]

S2 Fig. US County Map Showing Geographic Distribution of Sample Counties (n=2,096)[a]

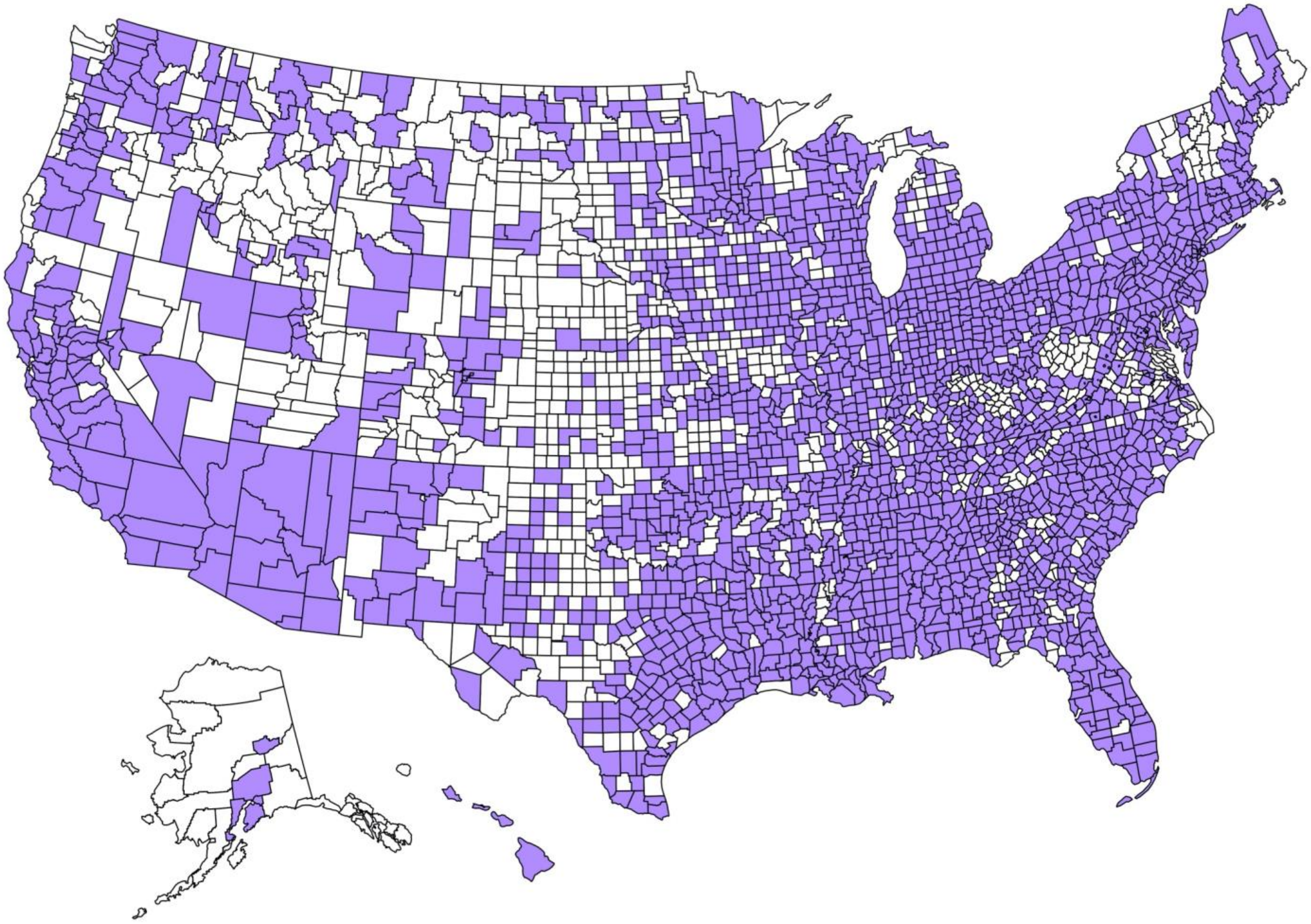

a. Map created using the usmap package in RStudio (https://CRAN.R-project.org/package=usmap)
