# Supplementary material for: COVID-19 and excess mortality in the United States: A county-level analysis
Source: PLoS Med. 2021 May 20;18(5):e1003571. doi: 10.1371/journal.pmed.1003571 (PMC8136644; doi:10.1371/journal.pmed.1003571)
Supplement: S4 Fig — (PDF) [file pmed.1003571.s004.pdf]

S4 Fig. Decomposition of 2020 Indirectly Age Standardized Excess Death Rates across Strata of Sociodemographic and Health Factors[a,b]

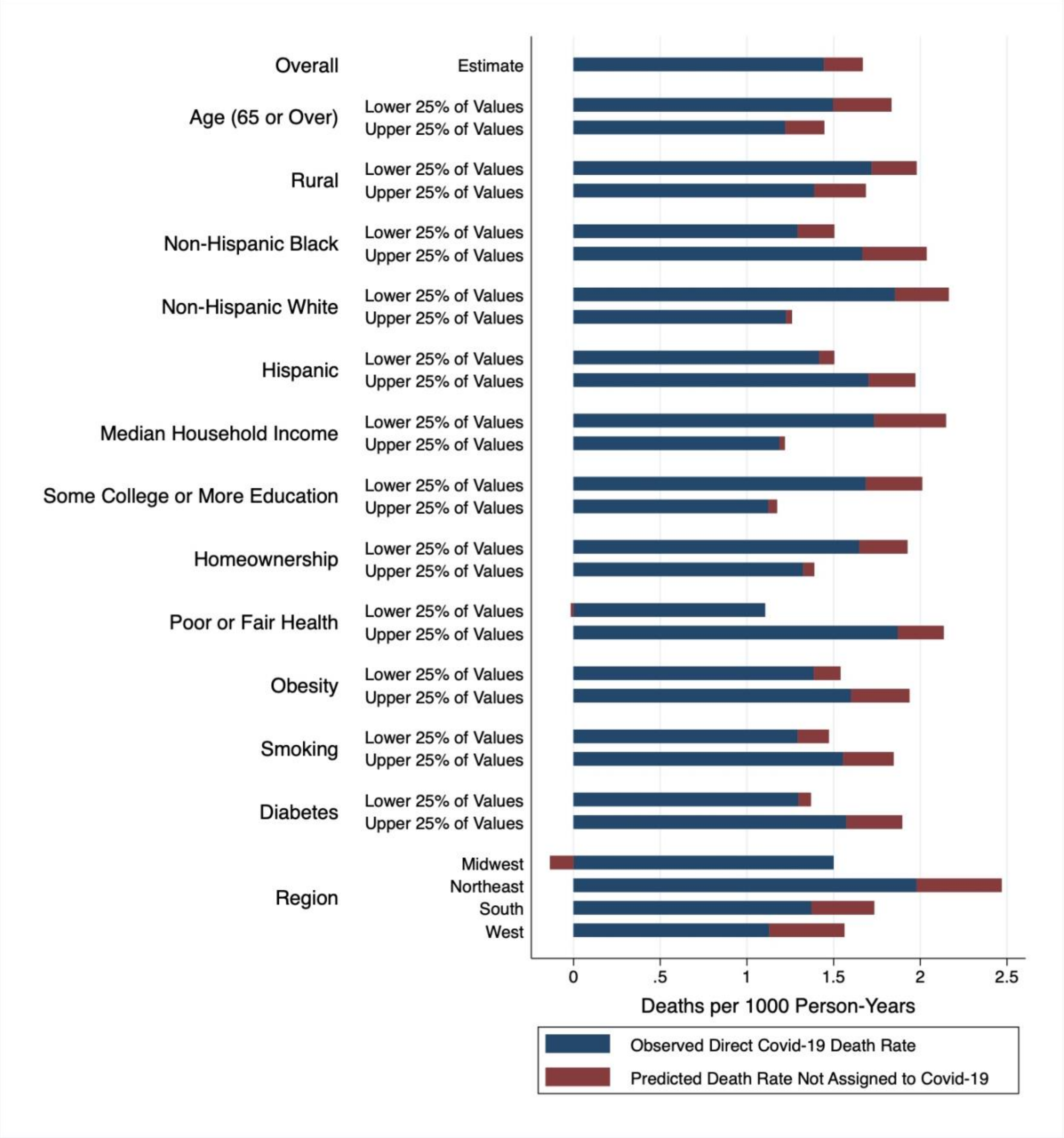

a. n = 2,096 counties

b. Predicted death rates generated from primary model: $M(i) = \alpha + \beta_1 M^*(i) + \beta_2 C(i)$, where $M(i)$ = Death rate from all causes in county i in 2020, $M^*(i)$ = Death rate from all causes, county i in 2013-2018, and $C(i)$ = Covid-19 death rate in county i in 2020. The model was weighted by the 2020 population and fully stratified into population weighted quartiles for each sociodemographic or health factor. The death rates for the upper and lower 25% of values for each factor are presented in this figure.
