# Supplementary material for: COVID-19 and excess mortality in the United States: A county-level analysis
Source: PLoS Med. 2021 May 20;18(5):e1003571. doi: 10.1371/journal.pmed.1003571 (PMC8136644; doi:10.1371/journal.pmed.1003571)
Supplement: S2 Table — (PDF) [file pmed.1003571.s006.pdf]

S2 Table. Data Sources and Years for County-Level Factors

**Variable**	**Data Source:**
% 65 Years and Older	Census Population Estimates, 2018
% Rural	Census Population Estimates, 2010
% Hispanic	Census Population Estimates, 2018
% Non-Hispanic Black	Census Population Estimates, 2018
% Non-Hispanic White	Census Population Estimates, 2018
Median Household Income	Small Area Income and Poverty Estimates, 2018
% with Some College or Higher	American Community Survey, 5-year estimates, 2014-2018
% Homeownership	American Community Survey, 5-year estimates, 2014-2018
% with Poor or Fair Health	Behavioral Risk Factor Surveillance System, 2017
% with Obesity	United States Diabetes Surveillance System, 2016
% who Smoke	Behavioral Risk Factor Surveillance System, 2017
% with Diabetes	United States Diabetes Surveillance System, 2016
