# Supplementary material for: COVID-19 and excess mortality in the United States: A county-level analysis
Source: PLoS Med. 2021 May 20;18(5):e1003571. doi: 10.1371/journal.pmed.1003571 (PMC8136644; doi:10.1371/journal.pmed.1003571)
Supplement: S3 Table — OLS, ordinary least squares. (PDF) [file pmed.1003571.s007.pdf]

S3 Table. Comparison of OLS, Indirectly Age Standardized and Negative Binomial Models

Model	Number of Excess Deaths per 1 Directly Coded Covid-19 Death	% Excess Deaths Not Attributed to Covid-19
OLS Model[a,b]	1.20 [95% CI (1.16, 1.24)]	17% [95% CI (14%, 19%)]
OLS Model, Age-Standardized[a,b,c]	1.15 [95% CI (1.12, 1.19)]	13% [95% CI (11%, 16%)]
Negative Binomial Model[d,e]	1.28	22%

a. The OLS models were specified as $M(i) = \alpha + \beta_1 M^*(i) + \beta_2 C(i)$, where $M(i)$ = Death rate from all causes in county i in 2020, $M^*(i)$ = Death rate from all causes, county i in 2013-2018, and $C(i)$ = Covid-19 death rate in county i in 2020. Model weighted by the 2020 population. For the Negative Binomial model, $M(i) =$ deaths from all-causes in county i in 2020 rather than the death rate, with the 2020 population used as an offset.

b. Number of excess deaths per 1 directly coded Covid-19 death is equivalent to the regression coefficient for directly coded Covid-19 deaths.

c. Death rates were indirectly age-standardized.

d. To calculate the number of excess deaths per 1 directly coded Covid-19 deaths, we used marginal prediction to calculate the all-cause death rate in 2020 at values of directly coded Covid-19 mortality that were +/- 0.1 deaths per 1000 people from the weighted mean of directly coded Covid-19 mortality. The change in all-cause mortality between these values was divided by 0.2 deaths per 1000 people to yield the number of excess deaths per 1 directly coded Covid-19 death.

e. A Poisson model was tested prior to the Negative Binomial model but was rejected due to poor goodness of fit.
