# Supplementary material for: COVID-19 and excess mortality in the United States: A county-level analysis
Source: PLoS Med. 2021 May 20;18(5):e1003571. doi: 10.1371/journal.pmed.1003571 (PMC8136644; doi:10.1371/journal.pmed.1003571)
Supplement: S4 Table — (PDF) [file pmed.1003571.s008.pdf]

S4 Table. Boundaries for Sociodemographic and Health Characteristic Quartiles[a]

Characteristics	Lower 25% Quartile		Upper 25% Quartile	
	**Lowest Value**	**Highest Value**	**Lowest Value**	**Highest Value**
% 65 or Older	7.4%	13.6%	17.6%	57.6%
% Rural	0%	1.3%	24.3%	100%
% Hispanic	0.6%	5.7%	26.0%	96.4%
% Non-Hispanic Black	0.1%	3.6%	18.6%	85.4%
% Non-Hispanic White	2.7%	42.1%	77.9%	97.9%
Median Household Income	25,385	52,577	74,686	140,382
% with Some College or Higher	20.4%	60.3%	71.8%	90.3%
% Homeownership	19.6%	56.9%	71.0%	89.8%
% Living with Poor or Fair Health	8.1%	14.0%	18.9%	41.0%
% with Obesity	14.4%	24.9%	32.8%	51.0%
% who Smoke	5.9%	12.6%	17.6%	41.5%
% with Diabetes	2.9%	8.4%	11.5%	34.1%

a. Quartiles are weighted by the estimated 2020 population.
